# High-Fidelity Simulation for Medical Students: Does Psychological Safety Increase During the Prebriefing Phase?

**DOI:** 10.7759/cureus.113533

**Published:** 2026-07-28

**Authors:** Timothy J Hodge, Janice W Kooken

**Affiliations:** 1 College of Osteopathic Medicine, Liberty University, Lynchburg, USA; 2 School of Education, Liberty University, Lynchburg, USA

**Keywords:** healthcare simulation, psychological safety, simulation in medical education, stress in simulation, team cohesion

## Abstract

The purpose of this quantitative, experimental pretest-posttest control group design was to examine whether the level of psychological safety a medical student brings to a high-fidelity simulation predicts their psychological safety and stress levels after a simulation event. Because recent research has found inconsistencies in the effectiveness of the prebriefing phase of a simulation learning event, this raises the question of whether a facilitator can adequately establish psychological safety during the prebriefing. This study sampled 114 students from a medical school in the mid-Atlantic region of the United States to examine if pre-existing psychological safety can predict postsimulation stress and psychological safety when using an intervention of psychological safety elements in the prebriefing and controlling for team cohesion. The pretest was taken one week prior to the simulation event, and the posttest was given immediately after the simulation activity and before the debriefing. To measure these items, this study used the Psychological Safety in High-Fidelity Simulation (PSHFS) scale, the Group Cohesion Scale (GCS), and the Perceived Stress Scale (PSS) through a combination of pretest and posttest electronic surveys. The data were analyzed using multiple regression and showed that pre-existing psychological safety scores were a significant predictor for both postsimulation psychological safety and stress scores. However, team cohesion and the treatment intervention were not significant predictors. This research suggested that the level of psychological safety a learner brings to a simulation may have a greater influence than previously thought. Future research should explore whether elements of providing psychological safety in the prebriefing are exchangeable based on a learner’s pre-existing psychological safety.

## Introduction

Psychological safety is considered an integral part of simulation for medical students, including its influence on team effectiveness and performance, yet there is disagreement over how medical simulation educators and scholars can establish it [1-3]. Turner and Harder [4] suggested that psychological safety could be established by using the elements of their concept analysis, but most research to date has not adequately addressed antecedent effects related to interpersonal experiences [3]. While recent research has called into question Rudolph’s Safe Container theory, numerous theories in simulation literature point to establishing psychological safety in the prebriefing [3,5].

In what is considered one of the seminal literature reviews and concept frameworks surrounding psychological safety in simulation, Rudolph et al. [5] suggested that simulation prebriefing is essential to creating a safe container for learners to explore the boundary conditions of their learning. However, the role, quality, and delivery of the prebriefing in contributing to positive outcomes remain inadequately understood [6]. Recent literature has called for “more quantitative data to better understand the direct effects of the delivery and quality of prebriefing on simulation outcomes and student experiences” [6]. Moreover, research is needed to explore “which teams are positioned to benefit most from simulation based on their pre-existing levels of psychological safety” and “understanding the dose and best participant targets to efficiently maximize impact” [3].

In this study, we examined the effect of an intervention using the Turner and Harder model criteria for delivering a prebriefing on perceived psychological safety and stress in a sample of medical students. The intervention reflected the necessary attributes for experiencing psychological safety [4]. We employed a pretest of psychological safety and group cohesion one week prior to the simulation to control for pre-existing differences in the participants. We examined possible differences in psychological stress and team cohesion measured after completion of the intervention and simulation.

While there are myriad elements that influence a student’s psychological safety, there appears to be a great deal of effort, focus, and research in the simulation environment on the prebriefing as the way to establish psychological safety. However, this seems overly simplistic for such a complex issue, and it likely explains the disparity in the research on this topic. Our methodology intentionally utilized a formative, low-stakes simulation activity for measurement to control for any threat to a student’s psychological safety or increase their stress from the simulation activity alone. This would permit the researchers to accurately measure a student’s arriving psychological safety or stress levels with only the prebriefing intervention and assist with controlling for confounds related to the simulation event itself. It could then be determined whether the student’s antecedent effects would change with the model criteria intervention on their perceived levels of psychological safety and stress. Similarly, the postsimulation measurements were taken prior to the debriefing to remove its potential influence on the model.

This article was previously posted to the Liberty University institutional repository as part of the doctoral dissertation of Timothy Hodge submitted on August 24, 2023.

## Materials and methods

The objectives of this study were to determine whether a model could be developed to use a student's perceived presimulation psychological safety, measured outside the simulation environment, to predict their postsimulation stress levels and postsimulation psychological safety. The model would determine whether a properly conducted prebriefing, as defined by the literature, could predict a student's postsimulation stress and psychological safety while controlling for team cohesion. To further evaluate the effectiveness of the intervention in the model, the researchers measured whether presimulation psychological safety itself could predict postsimulation stress levels and psychological safety. Therefore, the control groups were isolated for analysis. To meet these objectives, the researchers asked the following questions.

Research questions

The study addressed the following research questions to evaluate the predictive role of baseline perceived psychological safety and the impact of a psychological safety intervention on postsimulation stress and psychological safety among medical students participating in a high-fidelity simulation: (RQ1a) For medical students participating in a high-fidelity simulation, to what extent can the postsimulation stress level be predicted from pre-existing levels of perceived psychological safety for the control group? (RQ1b) For medical students participating in a high-fidelity simulation, what effect does a treatment providing elements of psychological safety have on postsimulation stress levels, controlling for pre-existing levels of perceived psychological safety and team cohesion? (RQ2a) For medical students participating in a high-fidelity simulation, to what extent can the postsimulation psychological safety level be predicted from pre-existing levels of perceived psychological safety for the control group? (RQ2b) For medical students participating in a high-fidelity simulation, what effect does a treatment providing elements of psychological safety have on postsimulation psychological safety, controlling for pre-existing levels of perceived psychological safety and team cohesion?

Methods

Design

The study was conducted at Liberty University College of Osteopathic Medicine on May 4, 2023. In this study, we used an experimental pretest-posttest control group design to test whether an intervention in the prebriefing would affect the postsimulation levels of stress and psychological safety over and above what was present prior to the simulation event. The intervention provided participants with all the strategies for establishing psychological safety in the prebriefing as defined by Turner and Harder [4]. Table 1 provides the treatment and control group components of psychological safety, and Appendices A and B provide the scripts.

**Table 1:** Strategies for establishing psychological safety The table is original and developed by the authors. The strategies were taken from Turner and Harder’s [4] concept analysis and used the model case criteria for the treatment group and the borderline case criteria for the control group.

Control group	Treatment group
Qualified facilitator	Qualified facilitator
Preparation	Preparation
Objectives and expectations	Objectives and expectations
-	Orientation
-	Ability to make a mistake without consequences

Measures

Perceived Stress Scale (PSS): The purpose of the PSS was to measure the perception of a situation as stressful, in this case after a high-fidelity medical simulation [7]. The original instrument was a 14-item scale [7]. It has undergone revisions, including a four-item scale and a shortened 10-item scale, but a review of the psychometric properties of all three scales showed the 10-item scale to be superior [8]. The PSS-10 was found to be as effective as the longer PSS-14 scale, yet it was determined to have a better factor structure and increased internal reliability [9]. Item numbers 1, 2, 3, 6, 9, and 10 measure perceived helplessness, and item numbers 4, 5, 7, and 8 measure a lack of self-efficacy [10,11]. The instrument is measured on a 0-40 scale, where higher scores represent higher levels of stress [10].

Group Cohesive Scale (GCS): Group cohesion is defined as a group working together for a common goal with mutual acceptance and identity with the group [12]. The purpose of the GCS was to measure cohesion and engagement, the only two elements in the original research that could predict group outcomes [13]. While this instrument was developed to study psychiatric patients with diminished motivation to participate in groups, the GCS measure was acceptable for any interactive group [13]. The GCS was developed by the medical community as a validated and reliable seven-item scale that could measure both cohesion and engagement, which are critical factors for healthcare simulation [5,13]. Group cohesion is a significant factor in fostering self-directed learning, and both are considered critical for medical students to be successful in medical school [12].

The GCS has not changed since its development as a seven-item scale where Questions 1-2 measure cohesion and Questions 3-7 measure engagement [13]. Importantly, the review of the GCS showed cohesion and engagement are likely similar in meaning and measurement, including factors with an extremely high correlation (r = 0.83), so the scale was measured as a total score instead of different subscales [13]. Scores were in the range of 7-35, with higher scores indicating greater group cohesion [13].

Psychological Safety in High-Fidelity Simulation Scale (PSHFS): The purpose of the PSHFS was to measure psychological safety in high-fidelity simulation learning environments to gain a deeper understanding of relationships between simulation design and psychological safety [14]. This scale was developed to replace the often-used Edmondson seven-item scale, which measures work environments but was never shown to be validated in the academic environment [14]. Although the PSHFS scale is relatively new, it directly measures the presence of psychological safety in a high-fidelity simulation, making it well suited for this research. More specifically, the scale measured the core attributes supported by other research for the measurement of psychological safety [4,14]. Scores range from 14 to 70, with higher scores representing higher levels of psychological safety in simulation by the student [14]. Questions 12 and 13 are reverse-scored because they had the opposite valence of the other questions.

Procedures

This study was submitted to the Institutional Review Board (IRB) of Liberty University for approval to conduct research with human subjects. After IRB approval was obtained (approval number: IRB-FY22-23-2021), the demographic questions and instruments were combined into a Qualtrics survey for online data collection. Once the survey was approved for distribution, a QR code and website were electronically generated for participants to access the survey.

This study used the concept analysis constructed by Turner and Harder [4], which provides a model and borderline criteria that provide the required elements to establish psychological safety in the prebriefing. For the current study, the treatment group received the elements outlined in the model case, and the control group received elements outlined in the borderline case (Table 1). The predictive model in the current study tested whether presimulation psychological safety had a stronger effect than providing all the elements outlined in the model case of the concept analysis.

After developing the treatment script for psychological safety, the researchers sought feedback from several simulation colleagues on the treatment’s capability to establish psychological safety. A jury of six subject matter experts met the criteria of knowledge of simulation operations, simulation education, and the use of psychological safety in simulation, of which two did not reply. The remaining four subject matter experts, consisting of professors and clinical simulation specialists, were asked to function as content validation jurors. The treatment script was modified based on the feedback received. Overall, the jurors agreed that the script provided psychological safety to participants.

One week prior to the simulation event, the researcher went to the lecture auditorium at the conclusion of the students’ patient-centered medicine lecture to provide the pretest. Participants received and signed a consent form prior to administration of the pretest. On the day before the simulation, four simulation rooms were set up and planned: two simulation rooms for the treatment groups and two for the control groups. All four simulation rooms would operate concurrently. The facilitators were randomly assigned to the simulation rooms to ensure that participant and facilitator pairing and facilitator assignment to lead treatment or control groups were by chance.

On the day of the simulation event, the researcher met with all the facilitators prior to the simulation to review how the facilitator script would serve as the entire prebriefing phase of the simulation. There were eight facilitators, who were experienced faculty who regularly participated in simulation and had all received the simulation program’s facilitator training. The facilitators were asked not to provide any additional details during the prebrief that were outside the script. Students arrived 15 minutes prior to the start time of their simulation event and were reminded of the research survey that would occur immediately after their simulation event. Once arriving at their simulation room, the facilitator greeted the students and began the prebrief. Both the control and treatment group facilitators read the prebriefing script verbatim. To ensure compliance by facilitators in delivering the script as outlined, the principal investigator routinely monitored facilitators providing the script throughout the study. This was accomplished by directly observing the delivery, and no deviations from the script were found. When the facilitator finished reading the script, the students were informed they could enter the simulation center to begin their simulation event. Students then participated in the simulation while the facilitator observed from the simulation control room. At the conclusion of the simulation, the facilitator met the students at the door to the simulation room and directed them to the debriefing room, where the students were administered the posttest (Table 2). Upon completion of the posttest, the facilitator re-entered the debriefing room and debriefed the simulation with the students and then dismissed them.

**Table 2:** Pretest and posttest instruments

Pretest instruments	Posttest instrument
Demographic questions	Demographic questions
Psychological Safety in High-Fidelity Simulation (PSHFS) scale	Perceived Stress Scale (PSS)-10
Group Cohesion Scale (GCS)	Psychological Safety in High-Fidelity Simulation (PSHFS) scale

Following all the simulation events and data collection, the raw data were exported from Qualtrics, imported into Excel (Microsoft Corp., Redmond, WA, USA) for cleaning and deidentification, and then uploaded into SPSS Statistics (IBM Corp., IBM SPSS Statistics for Windows, Armonk, NY). SPSS Statistics was used to analyze the data and perform multiple regression for the four research questions, generate frequency tables for the descriptive statistics, perform Pearson's correlations, and perform the post hoc paired t-test between pre- and postsimulation psychological safety. The data met all assumptions for conducting a multiple linear regression, including independence of residuals, linear relationships between the predictor and criterion variables, homoscedasticity of residuals, no multicollinearity, no significant outliers, and normally distributed residuals. Since the assumptions were met, a multiple regression was conducted for RQ1a, RQ1b, RQ2a, and RQ2b. The four hypotheses were tested using a significance level of α = 0.05.

Participants

The participants for this study came from a convenience sample of medical students enrolled in an osteopathic medical school during the 2022-2023 academic year. There were initially 121 participants who consented to the study, and 116 completed the pretest. After the conclusion of data gathering, 114 students had consented, completed the pretest, and completed the posttest. For this study, 114 participants achieved the desired level of significance and statistical power. Participation was voluntary, and students were allowed to participate in the simulation if they were not participating in the study.

For multiple regression, there was a 66-participant required minimum when assuming a medium effect size, statistical power of 0.7, and α = 0.05 [15]. Since the current study looked at data only for participants in the control group for two of the research questions, the total number of participants was increased to ensure sufficient statistical power remained for the control group. A post hoc power analysis was performed using G*Power version 3.1.9.7 (Heinrich Heine University Düsseldorf, Düsseldorf, Germany) to determine whether the study sample size met the required sample size to achieve 80% power to detect a medium effect at α = 0.05. The sample size of 114 for three predictor variables for a multiple regression was used to test RQ1b and RQ2b. The sample size of 52 for one predictor variable for a multiple regression was used to test RQ1a and RQ2a. The analysis resulted in a 0.99 power across all four research questions. Table 3 provides the descriptive statistics for the study.

**Table 3:** Frequency for sex, year in school, number of simulations, and group variables Due to rounding errors, percentages may not equal 100%. OMS 1: first-year osteopathic medical student, OMS 2: second-year osteopathic medical student

Variable	n	%
Sex		
Female	51	44.73
Male	63	55.26
Year in medical school		
OMS 1	114	100
OMS 2	0	0
Number of prior simulation events		
None	7	6.14
1-5	103	90.35
>5	4	3.51
Treatment and control groups		
Treatment group	62	54.39
Control group	52	45.61

Students were randomly assigned to the treatment and control groups by an Excel random number generator. For allocation concealment, participants were sorted via the random number generator into groups of three, from smallest to largest number. Then, each group was randomly assigned to a simulation room using the same method. The simulation rooms were assigned as a treatment or control group room using the random number generator. Then, the facilitator was also randomly assigned to a simulation room using this procedure. This procedure produced random assignment across participants, simulation rooms, treatment or control groups, and the facilitator.

Each group consisted of three participants for both the control and treatment groups. The control group received the partial psychological safety intervention, and the treatment group received the full psychological safety intervention. Comparing the control and treatment groups enabled model testing to determine whether the psychological safety intervention strategies changed stress levels or the perceived existence of psychological safety between the groups. The elements of the psychological safety intervention were implemented based on research by Turner and Harder [4], which stated that psychological safety’s defining strategies consisted of the qualities of the facilitator, the ability to make a mistake without consequences, and the foundational activities of orientation, preparation, and providing objectives and expectations. Both the control and treatment groups received a qualified facilitator, and students received the same clinical didactic and preparatory instruction prior to the simulation. The control group had 52 participants, and the treatment group had 62 participants. The final number of participants in each group changed based on participants consenting and completing both the pretest and posttest, which was required for inclusion in the study. A participant's data were removed from the study analysis if they failed to provide written consent, complete the pretest, or complete the posttest.

## Results

Data analysis

Descriptive statistics for each measure are provided in Table 4, and correlations between measures are provided in Table 5.

**Table 4:** Summary statistics for pretest and posttest instruments Only 114 participants were used in the analyses. n: sample size, Min: minimum, Max: maximum, M: mean, SD: standard deviation

Variable	n	Min	Max	M	SD
Pretest Psychological Safety in High-Fidelity Simulation (PSHFS)	114	23	69	47.39	9.03
Pretest Group Cohesion Scale (GCS)	115	8	35	28.15	4.56
Posttest Psychological Safety in High-Fidelity Simulation (PSHFS)	115	30	68	49.89	9.07
Posttest Perceived Stress Scale (PSS)-10	115	3	26	14.9	5.52

**Table 5:** Correlation for pretest and posttest instruments (n = 114) Pearson correlation, *p < 0.001, **p < 0.01

Variable	Pretest Psychological Safety in High-Fidelity Simulation (PSHFS)	Pretest Group Cohesion Scale (GCS)	Posttest Psychological Safety in High-Fidelity Simulation (PSHFS)	Posttest Perceived Stress Scale (PSS)-10
Pretest Psychological Safety in High-Fidelity Simulation (PSHFS)	1			
Pretest Group Cohesion Scale (GCS)	0.31*	1		
Posttest Psychological Safety in High-Fidelity Simulation (PSHFS)	0.75*	0.31*	1	
Posttest Perceived Stress Scale (PSS)-10	-0.51*	-0.22**	-0.50*	1

For RQ1a, multiple regression was used to predict postsimulation stress levels from presimulation psychological safety scores using only control group participants (n = 52). The model described by the regression equation was statistically significant, F (1, 50) = 25.44, p < 0.001, indicating a significant relationship between a participant’s presimulation psychological safety and their postsimulation stress level (Table 6). The Pearson correlation between presimulation psychological safety and postsimulation stress levels indicated a strong negative correlation, r(52) = -0.581, p < 0.001 [16]. The adjusted R² = 0.324, indicating that 32.4% of the variance in postsimulation stress levels could be explained by the model and was a medium effect size [16].

**Table 6:** Multiple regression results for stress level for control group - RQ1a Model: “enter” method in SPSS Statistics, B: unstandardized regression coefficient, CI: confidence interval for unstandardized regression coefficient, LL: lower limit, UL: upper limit, SE B: standard error of unstandardized coefficient, β: standardized coefficient. p < 0.001 for both the model intercept and the coefficient for presimulation psychological safety. F (1, 50) = 25.44, p < 0.001. n = 52; R² = 0.34 and Adj. R² = 0.32 (coefficient and adjusted coefficient of determination).

Postsimulation stress level	B	95% CI for B		SE B	β	t	p
		LL	UL				
Model							
Constant	29.01	23.42	34.61	2.78		10.42	<0.001
Presimulation psychological safety	-0.30	-0.41	-0.18	0.06	-0.58	-5.04	<0.001

For RQ1b, multiple regression was used to predict postsimulation stress levels by a participant’s presimulation psychological safety when examining the effect of a treatment for psychological safety during the prebriefing and on group cohesion. The model described by the regression equation was statistically significant, F (3, 110) = 12.90, p < 0.001, indicating that a participant’s presimulation psychological safety, group cohesion, and receiving psychological safety treatment during the prebriefing predicted their postsimulation stress level (Table 7). The adjusted R² = 0.240, indicating 24.0% of the variance in postsimulation stress level could be explained by the model and was a small effect size [16]. Only the coefficient for presimulation psychological safety was significant: treatment (β = -0.25, p = 0.78), group cohesion (β = -0.08, p = 0.45), and psychological safety pretest (β = -0.29, p < 0.001).

**Table 7:** Multiple regression results for stress level - RQ1b Model: “enter” method in SPSS Statistics, B: unstandardized regression coefficient, CI: confidence interval for unstandardized regression coefficient, LL: lower limit, UL: upper limit, SE B: standard error of unstandardized coefficient, β: standardized coefficient. p < 0.001 for the model intercept and the coefficient for presimulation psychological safety. p = 0.45 for group cohesion. p = 0.78 for treatment/control Group. F (3, 110) = 12.90, p < 0.001. n = 114; R² = 0.26 and Adj. R² = 0.24 (coefficient and adjusted coefficient of determination). The treatment/control group variable was dummy-coded, with the control group as the reference category (coded as 0) and the treatment group coded as 1.

Postsimulation stress level	B	95% CI for B		SE B	β	t	p
		LL	UL				
Model							
Constant	30.94	24.59	37.29	3.2		9.66	<0.001
Presimulation psychological safety	-0.29	-0.39	-0.19	0.05	-0.48	-5.57	<0.001
Group cohesion	-0.08	-0.29	0.13	0.1	-0.07	-0.77	0.45
Treatment/control group	-0.25	-2.04	1.54	0.9	-0.02	-0.28	0.78

For RQ2a, multiple regression was used to predict postsimulation psychological safety levels from presimulation psychological safety scores using only the control group participants (n = 52). The model described by the regression equation was statistically significant, F (1, 50) = 86.87, p < 0.001, indicating a significant relationship between a participant’s presimulation psychological safety and their postsimulation stress level (Table 8). The Pearson correlation between presimulation psychological safety and poststimulation psychological safety indicated a statistically significant, strong positive correlation, r(52) = 0.797, p < 0.001 [16]. The adjusted R² = 0.627, indicating 62.7% of the variance in postsimulation stress level could be explained by the model and was a large effect size [16].

**Table 8:** Multiple regression results for postsimulation psychological safety for control group - RQ2a Model: “enter” method in SPSS Statistics, B: unstandardized regression coefficient, CI: confidence interval of unstandardized coefficient, LL: lower limit, UL: upper limit, SE B: standard error of unstandardized coefficient, β: standardized coefficient. p < 0.001 for the coefficient for postsimulation psychological safety and p = 0.002 for the model intercept. F (1, 50) = 86.87, p < 0.001. n = 52; R² = 0.64 and Adj. R² = 0.63 (coefficient and adjusted coefficient of determination).

Postsimulation psychological safety	B	95% CI for B		SE B	β	t	p
		LL	UL				
Model							
Constant	12.71	4.79	20.64	3.94		3.22	0.002
Presimulation psychological safety	0.78	0.61	0.94	0.08	0.80*	9.32	<0.001

For RQ2b, multiple regression was used to predict postsimulation psychological safety from a participant’s presimulation psychological safety when examining the effect of a treatment for psychological safety during the prebriefing and group cohesion. The model described by the regression equation was statistically significant, F (3, 110) = 47.14, p < 0.001, indicating that a participant’s presimulation psychological safety, group cohesion, and receiving treatment for psychological safety during the prebriefing predicted their postsimulation psychological safety level (Table 9). The adjusted R² = 0.551, indicating 55.1% of the variance in postsimulation psychological safety level could be explained by the model and was a large effect size [16]. Only the coefficient for presimulation psychological safety was significant: treatment (β = 0.61, p = 0.60), group cohesion (β = 0.16, p = 0.23), and psychological safety pretest (β = 0.72, p < 0.001).

**Table 9:** Multiple regression results for postsimulation psychological safety - RQ2b Model: “enter” method in SPSS Statistics, B: unstandardized regression coefficient, CI: confidence interval of unstandardized coefficient, LL: lower limit, UL: upper limit, SE B: standard error of unstandardized coefficient, β: standardized coefficient. p < 0.001 for the coefficient for postsimulation psychological safety. p = 0.01 for the model intercept. p = 0.23 for group cohesion. p = 0.60 for treatment/control group. F (3, 110) = 47.14, p < 0.001. n = 114; R² = 0.56 and Adj. R² = 0.55 (coefficient and adjusted coefficient of determination). The treatment/control group variable was dummy-coded, with the control group as the reference category (coded as 0) and the treatment group coded as 1.

Postsimulation psychological safety	B	95% CI for B		SE B	β	t	p
		LL	UL				
Model							
Constant	10.81	2.66	18.96	4.11		2.63	0.01
Presimulation psychological safety	0.72	0.59	0.85	0.07	0.71	10.76	<0.001
Group cohesion	0.16	-0.1	0.43	0.13	0.08	1.22	0.23
Treatment/control group	-0.61	-1.69	2.9	1.16	0.03	0.52	0.60

## Discussion

Turner and Harder [4] suggested that all the components of their concept analysis were necessary to establish psychological safety; failing to provide all of them was likely to change a simulation participant’s perceived presence of psychological safety in the learning environment. Furthermore, the prebriefing phase has been assessed as a critical function in the simulation experience despite disagreement about its benefits, capabilities, and utility [17,18]. Kostovich et al. [19] expressed an urgent need for the prebriefing phase as the central element in establishing psychological safety’s foundation in a simulation learning event, while Dileone et al. [20] disagreed.

The results of RQ1a indicate that the pre-existing level of psychological safety measured one week prior to the simulation event was a significant predictor of postsimulation stress level under the condition of no intervention to improve psychological safety. This pre-existing psychological safety can be attributed to many factors beyond the scope of this research, including individual personality or the environment of the medical school outside the simulation event that day. The results of the regression analysis for this research question were that higher levels of pre-existing psychological safety predicted lower postsimulation stress levels for the control group. This suggests a change from the Turner and Harder [4] concept analysis, which purported that students who did not receive all elements of psychological safety during the prebriefing would be less likely to establish psychological safety. This finding does align with Purdy et al. [3], suggesting psychological safety is linked with real-world experiences rather than being limited to the simulation-based learning activity the student is about to undertake.

The purpose of RQ1b was to explore whether the experimental intervention was a significant predictor of stress when controlling for team cohesion and presimulation psychological safety. The current findings appear to differ from Turner and Harder’s [4] concept analysis. Prior research suggests that providing all the elements of psychological safety would improve participants' psychological safety [4,21,22]. One consideration to note may be that the dosage of psychological safety provided during the intervention may have been insufficient, or something within the inherent environment for the participants resulted in the intervention not being strong enough to change their postsimulation stress levels. However, the results showed that the pre-existing level of psychological safety predicted stress without the dosage of psychological safety provided by the intervention. As for team cohesion, Appelbaum et al.’s [23] research was supported because team cohesion and presimulation psychological safety were significantly correlated (r = 0.31, p < 0.001).

RQ2a sought to answer the extent to which postsimulation psychological safety could be predicted from pre-existing levels of perceived psychological safety for the control group. The results indicated that the pre-existing level of psychological safety a medical student had a week prior to a simulation event was a significant predictor of the postsimulation psychological safety level under the condition of no prebriefing. These results further call into question the benefits of the prebriefing phase of simulation, as they are not well-defined [17,18]. As explicated above, a medical student’s pre-existing level of psychological safety will be due to many factors outside the scope of this experiment. Nonetheless, inherent psychological safety does not diminish during a simulation event.

The purpose of RQ2b was to explore whether participants receiving all the elements of psychological safety would enhance the predictive model while controlling for team cohesion. We assumed that providing all these elements, given to the treatment group, during the prebriefing would improve participants' psychological safety [4,21,22]. We also tested whether team cohesion affected psychological safety in the predictive model [1]. While the overall model was significant, the intervention did not affect postsimulation psychological safety levels, differing from Turner and Harder’s [4] concept analysis of the model criteria. The results demonstrated that the level of psychological safety a participant enters with at the beginning of the simulation was predictive of their postsimulation psychological safety level. Although the intervention was not significant, once again we suggest that the medical school create a culture of sustained psychological safety above which the dosage was ineffective in changing postsimulation psychological safety.

To better understand the antecedents discussed, it may be helpful to review one element examined in this study: team cohesion. Due to the correlation, r(241) = 0.47, p = 0.01, between psychological safety and team cohesion found in Appelbaum et al. and the relationship established in this study for psychological safety and stress, it seemed intuitive that team cohesion may be a predictor of stress. Students facing the challenge of simulation as a team may improve their psychological safety. However, the failure of team cohesion to be a significant predictor for postsimulation stress level may not explain how team environments have improved both an individual’s self-efficacy and psychological safety in previous studies. Since participants were randomly assigned to their simulation groups, the overall intrinsic demands placed on them by participating in a simulation event may not have permitted team cohesiveness to adequately develop prior to the simulation event starting.

Although the intervention failed to cause a significant change between pre-existing and postsimulation psychological safety levels, certain elements may be more important for creating or increasing the dosage of psychological safety in different individuals. The dosage combined with the antecedent effects already discussed may result in a natural increase in psychological safety throughout the simulation learning event. There is evidence of this effect in this study where there was a significant increase in the mean of psychological safety from presimulation (M = 47.39, SD = 9.03) to postsimulation (M = 49.82, SD = 9.08). A post hoc paired-samples t test was completed to determine whether this increase was significant. The increase was significant (t = 4.025, p < 0.001) with a small effect (d = 0.377). These results occurred even though participants were in different simulation rooms, with different facilitators, and at different times of the day.

Limitations of this study included that all participants were first-year medical students with minimal prior simulation experience. Prior research has addressed the potential that repeated simulation exposure may assist with stress control [24]. The simulation experience was also formative rather than summative, and the simulation was a child wellness visit that likely provided no additional threats to the students' psychological safety. This effort was by design to test the antecedent effects but could be questioned with respect to whether the prebriefing could have changed the influence of such a threat in a perceived riskier simulation event. While facilitators were randomly monitored during the delivery of the scripts, the manner of delivery could have varied among facilitators in tenor, tone, or strict adherence to the words utilized between groups. Because the study was completed with a convenience sample from a single cohort of students from the same medical school and used self-reported surveys, the results could have some clustering effects. This raises concerns about generalization across simulation programs and healthcare settings. The authors sought to minimize these effects by increasing the sample size to ensure adequate statistical power; however, the study would benefit from further examination of the findings across different simulation programs and student populations.

## Conclusions

This research reflects the complex nature of psychological safety and the influence of antecedent effects on students participating in a simulation educational activity. Numerous factors consistently change a medical student’s overall psychological safety both inside and outside the academic environment. While generalization of the results across all simulation programs may be limited, psychological safety may or may not be changed by strongly supported prebriefing elements such as an experienced facilitator, type of activity, prework, or nature of the simulation. A standard prebriefing alone may not be enough to ensure every student’s psychological safety. General practices in simulation-based learning environments often maintain the prebriefing phase as a point when a safe environment is created. Consequently, best practices include a well-established preparation and prebriefing to set the stage for a psychologically safe environment. The current research illustrates that antecedents to the simulation experience may have a greater impact on psychological safety than the use of a prebriefing. For example, the overarching learning environment or the culture of their school may contribute substantially and therefore begin outside a simulation learning event.

The results suggested that antecedents of psychological safety may reduce stress without additional intervention from the facilitator. For example, a student’s sense of purpose in achieving their goals of medical school could be contributory and outside of the simulated environment. Aspects of the environment and culture of medical school, as well as personal attributes such as self-efficacy, team efficacy, personality, resilience, or even the individual’s current emotional state, may be strong determinants of psychological safety. Further, the state of psychological safety of classmates may influence an individual's level of safety despite strong safety measures put in place during the prebriefing phase. The antecedents establishing psychological safety outside the simulation center environment are very complex. Still, they are noteworthy as elements that may change psychological safety levels before the prebriefing phase and regardless of the threat that psychological safety is tested in the simulation activity. The practical application of this study suggests the learning environment and a student’s overarching level of psychological safety may have a greater impact on simulation-based education than previously thought. While adequate preparation and prebriefing of students is an important part of using simulation in medical education, best practices for prebriefing may not be holistic in establishing psychological safety. Additional student monitoring, measurement, and consideration of when simulation is utilized in curricular activities may be needed to account for antecedent effects on psychological safety. Future research will need to explore how overall learner environments affect a student’s psychological safety before a simulation-based educational event.

## Appendices

These scripts were developed by the authors following the suggested model and borderline case concepts from Turner and Harder [4].

Appendix A

Prebriefing Script - Strategies of Psychological Safety for the Control Group

Objectives and expectations

The objectives for this simulation are:

You will demonstrate the ability to take an appropriate pediatric history. You will demonstrate the ability to perform an appropriate pediatric examination. You will communicate effectively and work together as a team.

The expectation is that you will apply the information from your classes and labs into performing these skills and work together as a team to care for your patient.

Preparation

Do you have any questions about this clinical encounter?

Your roles during this simulation are: (facilitator will assign the history taker, physical examiner, and silent observer roles as noted on the schedule). Your roles and responsibilities are described on the back of your lanyard.

Scenario

In a pediatric clinic, you are asked to evaluate a six-year-old male patient who has come to the office today for his annual well visit. Please complete a history and physical exam. You will have 20 minutes to complete your simulation. If you finish early, please state to the patient that you will be leaving the room to speak to your attending. After completing your time, please return the room to the way you found it. We will then go to a separate area to conduct our debriefing.

Appendix B

Prebriefing Script - Strategies of Psychological Safety for the Treatment Group

Ability to make a mistake without consequences

Remember the fictional contract where you will treat this simulation as an actual clinical encounter. You cannot hurt the simulated patient simulator, and learning can come from the clinical encounter. There are no consequences for mistakes made during the clinical encounter.

Foundational activities

Orientation to the space

This is a simulated clinic where you will participate in your simulation. Hand sanitizer is on the wall, and a sink with hand-washing supplies is available to you. The dry-erase board is available for you and your partner(s) to take notes during the clinical encounter. The headwall at the head of the bed is available with suction and various oxygen delivery devices should you need them.

This is your patient. You may interact with the manikin as you would any patient. They will respond to you as appropriate for the clinical scenario. You may inspect the chest and abdomen as you would during an exam. You may auscultate heart, lung, and bowel sounds. If you need to expose the patient’s posterior, you should lie them flat and roll them on their side. You should communicate with the patient during this movement. The patient has palpable pulses and will have chest rise and fall with breathing.

Objectives and expectations

The objectives for this simulation are:

You will demonstrate the ability to take an appropriate pediatric history. You will demonstrate the ability to perform an appropriate pediatric examination. You will communicate effectively and work together as a team.

The expectation is that you will apply the information from your classes and labs into performing these skills and work together as a team to care for your patient.

Preparation

Do you have any questions about this clinical encounter?

Your roles during this simulation are: (facilitator will assign the history taker, physical examiner, and silent observer roles as noted on the schedule). Your roles and responsibilities are described on the back of your lanyard.

Scenario

In a pediatric clinic, you are asked to evaluate a six-year-old male patient who has come to the office today for his annual well visit. Please complete a history and physical exam. You will have 20 minutes to complete your simulation. If you finish early, please state to the patient that you will be leaving the room to speak to your attending. After completing your time, please return the room to the way you found it. We will then go to a separate area to conduct our debriefing.
